# Do Long-Term Conservation Pasture Management Practices Influence Microbial Diversity and Antimicrobial Resistant Genes in Runoff?

**DOI:** 10.3389/fmicb.2021.617066

**Published:** 2021-04-08

**Authors:** Yichao Yang, Amanda J. Ashworth, Lisa M. Durso, Mary Savin, Jennifer M. DeBruyn, Kimberly Cook, Philip A. Moore, Phillip R. Owens

**Affiliations:** ^1^Department of Crop, Soil, and Environmental Sciences, University of Arkansas, Fayetteville, AR, United States; ^2^USDA-ARS, Poultry Production and Product Safety Research Unit, Fayetteville, AR, United States; ^3^USDA-ARS, Agroecosystem Management Research Unit, Lincoln, NE, United States; ^4^Department of Biosystems Engineering & Soil Science, University of Tennessee, Knoxville, Knoxville, TN, United States; ^5^USDA-ARS, Nutrition, Food Safety/Quality, Office of National Programs, Beltsville, MD, United States; ^6^USDA-ARS, Dale Bumpers Small Farms Research Center, Booneville, AR, United States

**Keywords:** antimicrobial resistance, runoff microbiome, conservation agriculture, animal manure, microbial abundance

## Abstract

Runoff from land-applied manure and poultry litter is one mechanism by which manure-borne bacteria are transported over large distances in the environment. There is a global concern that antimicrobial resistant (AMR) genes may be transmitted through the food chain from animal manures to soil to surface water. However, details are lacking on the ecology of AMR genes in water runoff as well as how conservation management practices may affect the runoff microbiome or minimize the movement of AMR genes. The aim of this study was to identify microbial community structure and diversity in water runoff following 14-years of poultry litter and cattle manure deposition and to evaluate the amount of AMR genes under five conventional and conservation pasture management strategies. Since 2004, all watersheds received annual poultry litter at a rate of 5.6 Mg ha^−1^ and were consistently managed. Surface runoff samples were collected from each watershed from 2018 to 2019, characterized using Illumina 16S rRNA gene amplicon sequencing and enumerated for four AMR-associated genes (*ermB*, *sulI*, *intlI*, and *bla_ctx-m-32_*) using quantitative PCR. Overall, long-term pasture management influenced water microbial community structure, with effects differing by year (*p* < 0.05). Bacterial richness (Chao1 index) was influenced by pasture management, with the lowest richness occurring in the control (nearby non-agricultural water source) and the greatest under fields that were hayed (no cattle presence). Runoff bacterial richness in watersheds increased following poultry litter applications, indicating poultry litter is a possible source of bacteria and altered runoff community structure. The *bla_ctx-m-32_* gene was not detected in any surface water sample. The remaining three AMR genes were absent in the non-agricultural control, but present in agricultural samples. However, there was no impact (*p* > 0.05) from pasture management on the abundance of these genes, indicating both conventional and conservation practices have similar ecologies for these targets; however, there was a greater detection of *sulI* genes from runoff in continuously grazed systems in 2019, with hay being lowest in 2019. Results illustrate that the edge of field buffer strips may increase bacterial richness in water runoff, but these changes in richness do not greatly impact target AMR genes in the United States largest land-use category.

## Introduction

Livestock manure and byproducts are valuable fertilizer sources [namely nitrogen (N), phosphorus (P), and potassium (K)] in grassland systems, but their soil and water microbial ecologies may be affected by management. Previous work by Yang et al. (2019) evaluated how pasture management [hayed (H), continuously grazed (CG), rotationally grazed (R), rotationally grazed with a buffer strip (RB), rotationally grazed with a fenced riparian buffer (RBR), and a control (represented by nearby non-agricultural water samples)] affected soil bacterial diversity and found that CG systems had greater community richness, which corresponded with greater soil pH and nutrients. Consequently, continuously grazed systems reportedly increase soil microbial diversity, owing to continuous nutrient-rich manure deposition; however, this management strategy may adversely affect aboveground plant communities and water quality. In an additional study, Yang et al. (2020) quantified four antimicrobial resistant (AMR) genes in these soils after 14-years of continuous management and found that *ermB*, *sulI*, and *intlI* genes were the greatest under long-term CG (relative to the conservation best management practices), suggesting continuous cattle manure deposition may increase AMR gene presence. Therefore, soil is a natural reservoir of AMR bacteria and genes (Forsberg et al., 2012). However, other studies have indicated that AMR genes can be found in un-grazed and non-agricultural soils (Durso et al., 2012, 2016). Similarly, Cadena et al. (2018) identified tetracycline and sulfonamide antibiotic resistance genes in soils from organic farming operations.

In addition to nutrients, runoff from land-applied manures can carry bacteria and genes originating from both manure and soil. Large-scale rainfall events have been linked to decreases in microbial water quality, with 51% of waterborne disease outbreaks occurring following precipitation events (Hrudey et al., 2003; USEPA, 2007). Runoff may also contain AMR bacteria and genes (Barrios et al., 2020; Meyers et al., 2020). Following land application of poultry litter, antibiotics, AMR bacteria, and AMR genes may move from soil through runoff, leaching, and particle adsorbed runoff (Kay et al., 2004; Leal et al., 2013; Sun et al., 2013), thus potentially ending up in surface and groundwater (He et al., 2014). However, the extent of this is largely unknown in the United States largest land-use category.

Manure management practices have been shown to impact runoff. Kreuzig et al. (2005) found that litter incorporation reduced AMR concentrations in runoff relative to surface applications. Kay et al. (2004) identified that land application methods (i.e., chisel plowing compared to no-tillage) affected AMR surface water runoff. Thurston-Enriquez et al. (2005) also confirmed that bacterial and pathogenic fecal levels in runoff were elevated in a rainfall simulation study, which was attributed to manure land application. Numerous factors affect how soil and fecal microorganisms are transported in manure-amended fields, although little is known about how specific management practices (e.g., rotational grazing) affect runoff microbial communities or the composition of AMR genes. Conservation management practices may help to mitigate AMR gene distribution (Heinonen-Tanski and Uusi-Kamppa, 2001; Tate et al., 2006), although the degree of this is unknown.

Here, we test the hypothesis that pasture management will impact the runoff microbial communities of long-term watersheds (14-years) receiving poultry litter amendments and determine if conservation agricultural practices can minimize the dissemination of four AMR genes chosen to represent targets important in human health, agriculture, and environmental AMR surveillance.

## Materials and Methods

### Treatment Implementation and Sample Collection

A field study was initiated in 2004 at the USDA-ARS Dale Bumpers Small Farms Research Center in Booneville, Arkansas (N 35°06'12'' W 93°56'05'' 150 m altitude) to evaluate the impact of conservation pasture management on water quality (Pilon et al., 2017a,b, 2018; Yang et al., 2019, 2020; Anderson et al., 2020). Fifteen watersheds were constructed on a site with an average slope of 8% and on an Enders (fine, mixed, active, and thermic Typic Fragiudults) and Leadvale silt loam (fine-silty, siliceous, semiactive, and thermic Typic Fragiudults). Each watershed was 25 × 57 m for a total area of 0.14 ha, where common bermudagrass (*Cynodon dactylon* L.) was the dominant forage.

**Figure 1:**
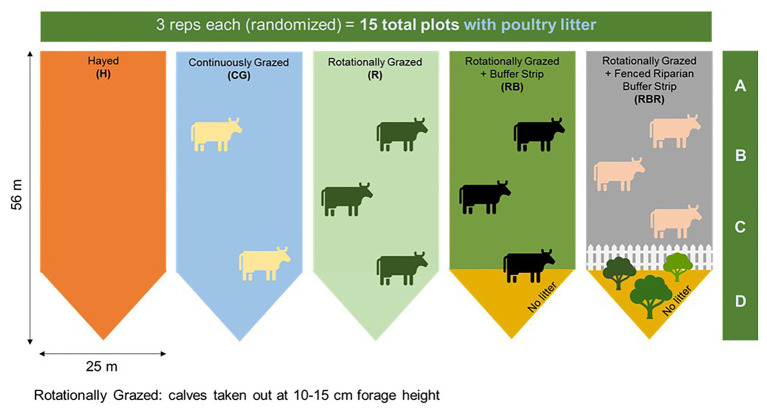
Schematic representation of the experimental set-up. Randomized complete block design with three replications (15 watersheds total) from 2004 to 2019. All areas have received annual poultry litter applications. Figure credit: Amorim et al. (2020). Continuous treatments included: hayed (H), continuously grazed (CG), rotationally grazed (R), rotationally grazed with a buffer strip (RB), and rotationally grazed with a fenced riparian buffer (RBR). Water samples were collected from 2018–2019 at Booneville, AR. For both years, both pre and post samples were used in 16S sequencing (*n* = 60 total), but only post samples were used in qPCR analyses (*n* = 30 total). Therefore, only 16S methods evaluated poultry litter timing effects.

Four agricultural management strategies were evaluated, along with one non-agricultural set of control samples. Grazing management strategies were implemented from 2004 to 2019 with three replications: hayed (H), continuously grazed (CG), rotationally grazed (R), rotationally grazed with a buffer strip (RB), and rotationally grazed with a fenced riparian buffer (RBR; Figure 1). The H treatment was hayed three times annually (April, June, and October) to a height of 10 cm with a rotary hay mower (no cattle in these watersheds). The CG watersheds were continuously grazed by one or two calves throughout the year. The R watersheds were rotationally grazed by three steers turned into the paddocks when forage height was 20–25 cm (10–15 cm using a disc meter) and removed when forage height was 10–15 cm (5 cm using disc meter). The RB watersheds were rotationally grazed with a 15.3-m buffer strip composed of the same vegetation (total area of 283 m^2^) at the base of these watersheds. The RBR watersheds were rotationally grazed with a fenced riparian buffer area to exclude cattle and planted with four sapling white oak (*Quercus alba* L.), four green ash (*Fraxinus pennsylvanica* Marshall), and four pecan [*Carya illinoinensis* (Wangenh.) K. Koch] trees in 2003 (Pilon et al., 2017a; Figure 1). Each watershed was divided, perpendicular to the slope into three zones (corresponding to shoulder, upper backslope, and lower backslope positions), whereas the RBR consist of four zones. Broiler litter was land applied at a rate of 5.6 Mg ha^−1^ in April or May of each year to each watershed (excluding the riparian buffer strip). Since poultry litter was omitted in the buffered area of the RB and RBR treatment, application rates were identical on an aerial basis (RB and RBR watersheds received 658 kg plot^−1^, whereas H, R, and CG received 794 kg plot^−1^). Broiler litter was obtained annually from a commercial broiler farm near Booneville, AR.

Watersheds were hydrologically isolated from each other by earthen berms that were constructed with offsite soil. Briefly, the bottom of each watershed narrows to a point containing a covered 30.5-cm H-series fiberglass flume equipped with a pressure transducer for measuring runoff volumes (Pilon et al., 2017a). The transducer was connected to a housed automatic water sampler (American Sigma Corporation), which was programed to automatically collect 100 ml of sample for analysis from every 94.7 L of surface runoff. Surface runoff water samples (one per watershed) were collected after each rain event (typically within 24 h). Runoff samples collected during 2018 and 2019 were utilized for this study. For both years, there were 60 water runoff samples [30 runoff samples collected 3 months prior to poultry litter application (“pre”), and 30 runoff samples collected within 3 months following poultry litter application (“post”)]. For both years, both pre and post samples were used in 16S sequencing (*n* = 60 total), but only post samples were used in qPCR analyses (*n* = 30 total). Therefore, only 16S methods evaluated poultry litter timing effects.

Since all watersheds used in this study received animal inputs (poultry litter and cattle manure), water samples from a more pristine water source (the Mulberry River) were included to serve as a control. Evaluation of AMR in non-agricultural samples provides background data on AMR occurring in similar soils without agricultural impacts. These background data are used to contextualize study results and provides insight on the impact of agricultural management practices on AR within agroecosystems (Rothrock et al., 2016). The Mulberry River is a 110 km long tributary of the Arkansas River in northwest Arkansas and has been designated as a National Wild and Scenic River since 1992. Samples were collected (Latitude: 35.6693 Longitude: −93.6676; *n* = 3 per year) and stored as described above.

### DNA Extraction, qPCR Amplification, and Sequencing

Each water sample was filtered by placing a sterile member filter (45 mm, 0.45 μm pore size, polycarbonate) on the filter base, grid-side up, and then placing another filter (47 mm, 1.2 μm, cellulose) on top of the 0.45 μm polycarbonate filter. After 250 ml of water passed through, filters were removed from the filter base and sterile forceps were used to aseptically discard the 1.2 μm filter. The 0.45-μm isopore filter was folded and transferred to corresponding labeled Lysing Matrix E tube. DNA was extracted from each water sample using the extraction kit of MpBio FastDNA Spin Kit for Soil (MpBio Laboratories, SKU 116560200-CF) according to the manufacturer’s directions. Extracted DNA was quantified using Quant-It™ PicoGreen® (Invitrogen) dsDNA quantitation assay and stored at 20°C.

Bacterial community composition was determined using Illumina Miseq sequencing of 16S rRNA gene amplicons. Extracted DNA was sent to the University of Tennessee Genomic Services Laboratory, where the V4 region of the 16S rRNA gene was amplified with barcoded primers 515F and 806R (Caporaso et al., 2011). Amplicon libraries were pooled and 291 base-paired end sequences were obtained on the Illumina MiSeq Platform, resulting in a total of 5,997,907 sequence reads. Reads were processed using the open source bioinformatics software Mothur V 1.40.0 following the Miseq SOP protocol (Kozich et al., 2013). Sequences that did not match the primers were eliminated from demultiplexed sequence reads. These ambiguous base sequences with a length less than 100 bp were deleted and chimeric sequences were removed using the UCHIME algorithm implemented in Mothur. After the quality control pipeline, 5,969,039 sequence reads remained using a 97% similarity threshold to define ribotypes in Mothur (21.27% were deleted). Taxonomic assignment was performed using the Greengenes database. Microbial alpha diversity in observed operational taxonomic unit (OTU) level including Chao1, Shannon index, and Simpson index were calculated using Mothur. To detect significant differences based on fixed effects of pasture management (H, CG, R, RB, and RBR), timing of sampling (pre or post poultry litter application), and random effects (year and replication), an analysis of variance (ANOVA) was used by the JMP software (JMP®12; SAS Institute, 2007). Probability values less than 0.05 were considered as significant. Beta-diversity was measured using Bray-Curtis index. Principle coordinate analysis (PCoA) plots were generated based on weighted and unweighted UniFrac distance metrics by using MicrobiomeAnalyst (Dhariwal et al., 2017). Bacterial community structure was quantified in a matrix of Bray-Curtis similarities, which was then analyzed in a permutational analysis of variance (PERMANOVA) to compare bacterial communities at the phylum level in PRIMER-E.

### Detection and Analysis of Four AMR Genes Following Long-Term Management Using qPCR

The extracted DNA from runoff following poultry litter applications (*n* = 30; 15 from October of 2018 and the other 15 from October 2019) were used directly in the quantitative PCR (qPCR) for detection of four genes associated with AMR described in clinical isolates, which includes *ermB* (Florez et al., 2014), *sulI* (Barraud et al., 2010), *intlI* (Pei et al., 2006), and *bla_ctx-m-32_* (Szczepanowski et al., 2009), using previously published primers (Yang et al., 2019). Each PCR amplification was performed in triplicate. The positive control (named as gBlock2 4G with 16S *ermB* Florez 1-18-17) is an 808 bp double stranded synthetic gBlocks® gene fragment synthesized by Integrated DNA technologies, lnc. (Blazejewski et al., 2019). It contains four genes of *ermB*, *sulI*, *intlI*, and *bla_ctx-m-32_*. The standard curves consisted of a serial dilution of known copy numbers of the gene fragment, ranging from 1.15 × 10^5^ to 1.15 × 10^11^ copies per 5 μl. The quantities of gene copy numbers were calculated based on the standard curve using Quant Studio 3 real-time PCR system. (ThermoFisher Scientific). As a negative control, all sets of primers were tested with sterile water as the template, and all of them were below threshold. Each 20 μl qPCR reaction included 5 μl of extracted DNA (approximately 100 ng), 10 μl of SYBR Green PCR Master Mix, and 100 mM of each primer. The following cycling conditions were used: an initial denaturation step of 15 min at 95°C, followed by 40 cycles of 15 s at 95°C, 30 s at annealing temperature specific for each gene (Yang et al., 2019), and 10 s at 72°C, followed by 60–95°C of melting curve. The amplification efficiency was between 92 and 105%, and the *R*^2^ value is above 0.98. Baseline and threshold calculations were performed using QuantStudio® Design & Analysis software. The quantities of gene copy numbers were determined using standard curves. Gene copy abundances were normalized per volume of water. Finally, the gene copy numbers per volume were transformed into log10 values for further statistical analysis, as they were not normally distributed (Ganger et al., 2017). To detect significant differences for the fixed effect (pasture management) and random effect (year), an ANOVA was used by the JMP software (JMP®12; SAS Institute, 2007), with replicate as a random effect. Probability values less than 0.05 were considered significant.

This set of targets was chosen to cover clinically relevant, environmentally relevant, and agriculturally relevant antibiotic resistance determinants. The specific targets were chosen by a panel of scientists working on antibiotic resistance in agriculture, and they aligned with an environmental antibiotic resistance gene surveillance effort in Europe. The blaCTX-M gene codes for third-generation cephalosporin resistance, one type of β-lactamase resistant drug. These drugs are classified as “Critically Important” (the top category) by WHO. Most individual drugs in the class are limited to use on humans, pets (dog/cat), and horses; however, two (cefquinome and ceftiofur) are indicated for use in food animals, though they are not administered to groups of animals *via* food or water. The ermB gene codes for resistance to macrolide drugs such as erythromycin. These drugs are classified as “Critically Important” (the top category) by the WHO. Erythromycin is used in large and small animals, is FDA approved for use in cattle, swine, and poultry, and is administered to food animals *via* food and water. A related macrolide, azithromycin, is also approved for use in sick food animals and is individually listed on the 2013 CDC AR Threat list as a concern for some foodborne pathogens. The sul1 gene codes for sulfonamide-resistance. It is one of the most commonly studied resistance genes in environmental samples. Sulfonamides are classified as “Highly Important” (the second category) by the WHO. Three drugs in this class are used in food animals and administered to groups of animals *via* food and water. Finally, the intl1 gene codes for an integron-integrase gene that helps AR genes to spread from cell to cell. It has been proposed as a gene that will help to identify resistance that is associated with human activities, and as a marker for “pollutants” including AR, heavy metals, and disinfectants.

## Results

### Effects of Pasture Management, Poultry Litter Application Timing, and Sampling Year on Alpha Diversity of Runoff Bacterial Communities

#### Comparisons of Runoff Among Pasture Management Systems (Control Excluded)

**Figure 2:**
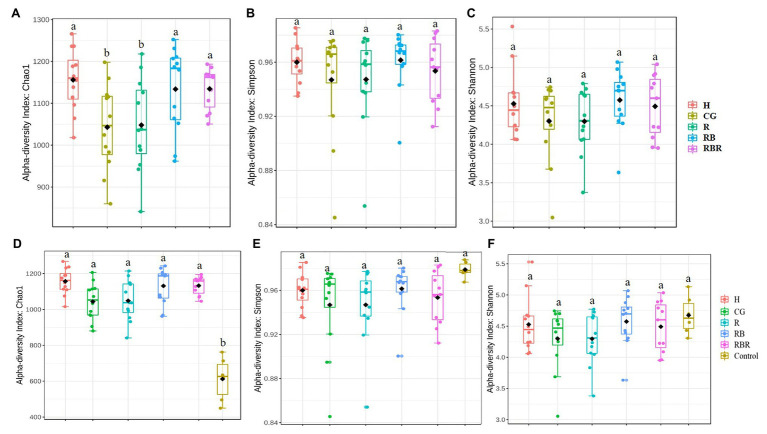
Alpha diversity of bacterial communities in runoff water from different pasture management treatments. Treatments included: hayed (H), continuously grazed (CG), rotationally grazed (R), rotationally grazed with a buffer strip (RB), and rotationally grazed with a fenced riparian buffer (RBR). Illustrates results from only pasture management included three indices of Chao1 **(A)**, Simpson **(B)** and Shannon **(C)**. The factor includes non-agricultural samples (control), Chao1**(D)**, Simpson **(E)**, and Shannon **(F)**.

Control samples were excluded in analyses to evaluate how agricultural practices influenced explanatory variables (within watersheds) but are included in the next section. Conservation pasture management including grassed buffer strips and riparian buffers affected bacterial richness (Chao1 estimate), with greater richness occurring in H, RB, and RBR and the lowest richness in CG and R (*p* < 0.05; Table 1; Figure 2A). However, there was no impact on bacterial diversity estimated by Shannon and Simpson indexes, when control samples were excluded in the analysis (*p* > 0.05; Figures 2B,C; Table 1). Bacterial richness increased in runoff following poultry litter application (*p* < 0.05). However, poultry litter application timing had no impact on runoff bacterial diversity (*p* > 0.05; Table 1). There was an interaction between year and poultry litter application timing on bacterial richness, with pre-poultry litter applications in 2018 being the lowest and post applications in 2019 being the greatest (*p* < 0.05; Table 1). Although there was no year effect on richness (*p* > 0.05), diversity varied by year (*p* < 0.05; Table 1). Overall, runoff bacterial diversity was lower in 2018 and greater in 2019 (Table 1).

**Table 1:** ANOVA of richness (Chao1) and diversity (Shannon) in alpha diversity influenced by pasture management and year following 13 years of pasture management and year.

	Parameter	Factor	*df*	*F*-value	*p*
With Control Samples	Richness	Pasture management^⸸^	5	3.1464	0.0235^*^
		Timing	1	0.0884	0.7673
		Year	1	0.262	0.6106
		Pasture management × Year	5	0.7954	0.5347
		Pasture management × Timing	5	2.7036	0.0428^*^
		Year × Timing	1	3.6696	0.0601
		Pasture × Year × Timing	5	1.1895	0.3291
	Diversity	Pasture management	5	1.1805	0.3329
		Timing	1	1.08	0.2854
		Year	1	4.9233	0.0302^*^
		Pasture management × Year	5	0.1155	0.9763
		Pasture management × Timing	5	1.2247	0.3145
		Year × Timing	1	0.0304	0.862
		Pasture × Year × Timing		0.8642	0.4931
Without Control Samples	Richness	Pasture management	4	2.9211	0.0332^*^
		Timing	1	1.2636	0.0005^*^
		Year	1	0.0988	0.7549
		Pasture management × Year	4	0.7385	0.5715
		Pasture management × Timing	4	2.5099	0.0573
		Year × Timing	1	6.1145	0.0176^*^
		Pasture × Year × Timing	4	1.1043	0.3683
	Diversity	Pasture management	5	1.0732	0.3829
		Timing	1	1.0907	0.3028
		Year	1	9.7051	0.0034^*^
		Pasture management × Year	5	0.1050	0.9801
		Pasture management × Timing	5	1.1134	0.3641
		Year × Timing	1	0.0238	0.8782
		Pasture × Year × Timing	5	0.7856	0.5415

⸸ Treatments included: hayed (H), continuously grazed (CG), rotationally grazed (R), rotationally grazed with a buffer strip (RB), rotationally grazed with a fenced riparian buffer (RBR); Timing = poultry litter application timing, pre and post; Year = 2018 and 2019.

* *p* < 0.05.

To evaluate differences in alpha diversity between water runoff samples under pasture management and the control, a comparison between these two datasets was conducted. Bacterial richness in control samples were lower than samples collected from watersheds under pasture management (*p* < 0.05; Figure 2D). However, there were no diversity differences between control samples and pasture management based on Shannon and Simpson indices (Table 1; Figures 2E,F). Year also had no influence on bacterial richness, however, there was an impact of year on bacterial diversity between runoff and control samples (*p* < 0.05). An interaction between pasture management and poultry litter application timing (pre vs. post) on richness was also detected, with pre in the control being lowest and post in the H treatment the greatest (*p* < 0.05; Table 1).

### Bacterial Community Composition Based on Pasture Management, Poultry Litter Application Timing, and Sampling Years

**Figure 3:**
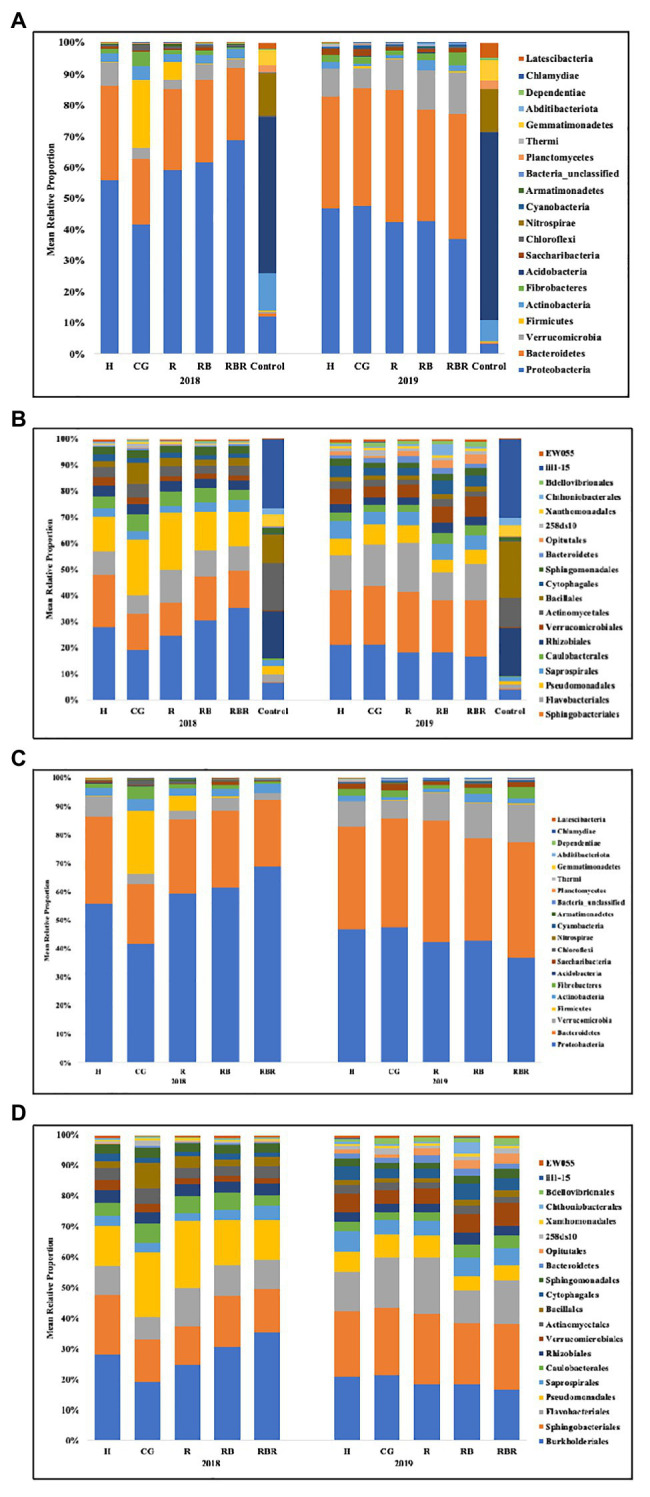
Mean relative proportion of bacterial taxa at the phylum **(A)** and order **(B)** level found in runoff (with or without control samples included in the analysis) following long-term conservation pasture management. Treatments included: hayed (H), continuously grazed (CG), rotationally grazed (R), rotationally grazed with a buffer strip (RB), and rotationally grazed with a fenced riparian buffer (RBR). **(A)** Bacterial community composition at the phylum level found in runoff with control samples. **(B)** Bacterial community composition at the order level found in runoff with control samples. **(C)** Bacterial community structure in phylum level found in runoff without control samples. **(D)** Bacterial community structure in order level found in runoff without control samples.

Runoff water bacterial community composition was not altered based on pasture management (H, CG, R, RB, and RBR; *p* > 0.05) at the OTU or order level (Table 2; Figure 3). However, there was a difference in bacterial community composition between water runoff and control samples at both levels (OTU and order; PERMANOVA *p* < 0.05; Table 2; Figure 3B). Year also had an impact on bacterial communities (with or without control samples; Table 2; Figure 3), as it differed in water bacterial communities at the phyla level between sampling years (2018 and 2019; *p* < 0.05; Figure 3).

**Table 2:** PERMANOVA in bacterial community structure by pasture management and year with or without non-agricultural samples (control).

	Factor	Pseudo-F	*p*
Including Control Samples	Pasture management^⸸^	0.8921	0.0095^*^
(OTU level)	Year	7.1399	0.0001^*^
	Pasture management × Year	−0.6100	0.7671
Including Control Samples	Pasture management	11.871	0.0001^*^
(Order level)	Year	12.048	0.0001^*^
	Pasture management × Year	0.4976	0.5596
No Control Samples	Pasture management	0.8184	0.5156
(OTU level)	Year	7.3945	0.0001^*^
	Pasture management × Year	−1.0893	0.5358
No Control Samples	Pasture management	0.6904	0.701
(Order level)	Year	12.532	0.0001^*^
	Pasture management × Year	−1.1535	0.6112

⸸ Treatments included: hayed (H), continuously grazed (CG), rotationally grazed (R), rotationally grazed with a buffer strip (RB), rotationally grazed with a fenced riparian buffer (RBR); Year = 2018 and 2019.

* *p* < 0.05.

The bacterial community composition was analyzed at two different levels (phylum and order). The following top 10 phyla dominated agricultural runoff bacterial communities: Proteobacteria (mean relative abundance of all libraries was 50.7%), Bacteriodetes (30.2%), Verrucomicrobia (6.4%), Firmicutes (3.1%), Actinobacteria (2.7%), Fibrobacteres (2.0%), Acidobacteria (1.2%), Saccharibacteria (TM7; 0.9%), Chloroflexi (0.6%), and Nitrospirae (0.3%; Figure 3A). The following top 10 orders were Burkholderiales (22.9%), Sphingobacteriales (18.1%), Flavobacteriales (11.8%), Pseudomonadales (11.5%), Saprospirales (4.4%), Caulobacterales (4.1%), Rhizobiales (4.0%), Verrucomicrobiales (3.9%), Actinomycetales (3.5%), and Bacillales (3.2%; Figure 3B).

**Figure 4:**
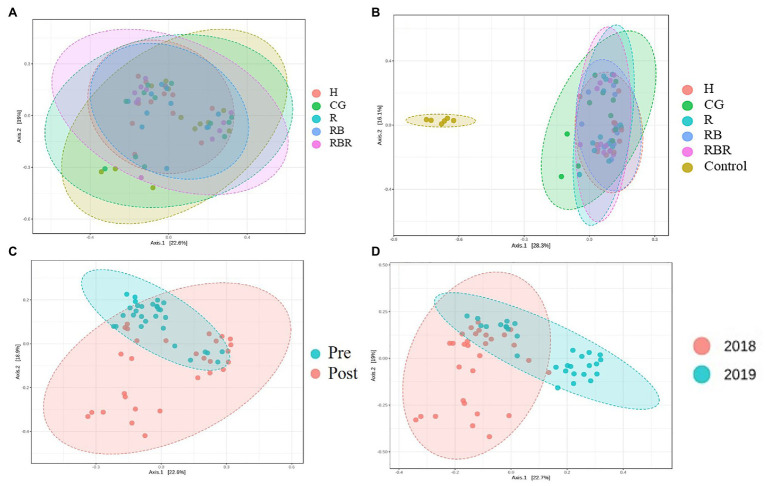
The beta diversity of bacterial community impact by pasture management [without **(A)** or with control samples **(B)**], sampling collection timing (pre and post poultry litter surface application; C) and year (2018 and 2019; D) in water samples. Treatments included: hayed (H), continuously grazed (CG), rotationally grazed (R), rotationally grazed with a buffer strip (RB), and rotationally grazed with a fenced riparian buffer (RBR).

PCoA of Bray-Curtis distance of the bacterial community visualized differences between pasture management systems and non-agricultural samples (control). However, the bacterial community composition was not influenced by pasture management (when pristine samples were excluded from metadata), suggesting there was no difference among H, CG, R, RB, and RBR (Figure 4A) in terms of runoff water bacterial communities. However, the bacterial community composition was different between pasture management and control samples (Figure 4B).

Beta diversity was influenced by poultry litter application timing, with greater beta diversity in runoff samples collected after poultry litter application and lower diversity in runoff samples collected before poultry litter application (Figure 4C). Year also had an influence on the beta diversity of runoff, resulting in greater diversity in 2018 and lower diversity in 2019 (Figure 4D).

### Distribution of Four AMR Associated Genes in Runoff Based on Pasture Management and Sampling Time

#### Quantification of ARG Targets

**Figure 5:**
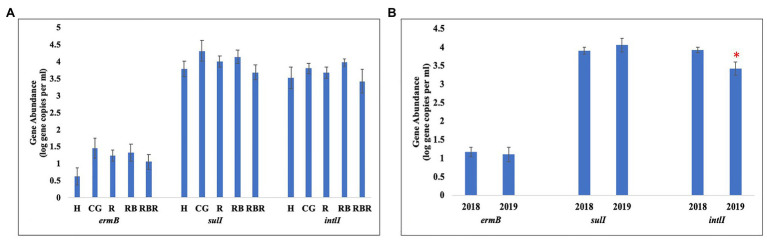
Mean abundances of three AMR associated genes, *ermB*, *sulI*, and *intlI* amplified from water genomic DNA samples based on (A) pasture management (H, CG, R, RB, and RBR), (B) sampling year (2018 and 2019). Treatments included: hayed (H), continuously grazed (CG), rotationally grazed (R), rotationally grazed with a buffer strip (RB), and rotationally grazed with a fenced riparian buffer (RBR). Error bars represent standard errors. The star indicates a significant difference at an alpha level of 0.05.

Pasture management (H, CG, R, RB, and RBR) and year (2018 and 2019; *P* > 0.05) had no impact on the abundance of *ermB* gene (Table 3; Figure 5), nor was there an interaction for pasture management × year on the abundance of *ermB* genes (*p* > 0.05). The *bla_ctx-m-32_* gene was not detected in any water runoff samples, and *bla_ctx-m-32_* was therefore not included in Tables or Figures. Similarly, there was no amplification of the four AMR-associated genes from the control water samples.

**Table 3:** ANOVA results testing for differences in quantities of three AMR genes following poultry litter soil applications (runoff samples collected within 3 months) without control samples by pasture management, as well as the interaction between these two factors at Booneville, AR from 2018 to 2019.

Parameter	Factor	df	F-value	*p*
*ermB*	Pasture management^⸸^	4	1.74	0.17
	Year	1	0.08	0.78
	Pasture management × Year	4	0.52	0.72
*sulI*	Pasture management	4	1.30	0.30
	Year	1	0.54	0.47
	Pasture management × Year	4	2.98	0.04^*^
*intlI*	Pasture management	4	0.83	0.52
	Year	1	6.76	0.01^*^
	Pasture management × Year	4	1.45	0.25

⸸ Treatments included: hayed (H), continuously grazed (CG), rotationally grazed (R), rotationally grazed with a buffer strip (RB), and rotationally grazed with a fenced riparian buffer (RBR); Year = 2018 and 2019.

* *p* < 0.05.

Pasture management (H, CG, R, RB, and RBR) had no impact on the abundance of *intlI* gene (*p* > 0.05), however, differences in abundance of *intlI* gene was identified between 2018 and 2019, with a greater abundance of *intlI* occurring in 2018 than 2019 (*p* < 0.05; Figure 5). There was no interaction for pasture management × year on the abundance of *intlI* genes (*p* > 0.05). Similarly, there was no difference in *sulI* gene detection based on pasture management (H, CG, R, RB, and RBR), and year (2018 and 2019; *p* > 0.05). However, the interaction between pasture management and year had an influence on the abundance of *sulI* genes detected from runoff samples (*p* < 0.05; Table 3). Overall, CG was the greatest in 2019 and H being the lowest in 2019.

## Discussion

### Effect of Pasture Management, Poultry Litter Application, and Year on Alpha Diversity of Water Runoff Bacterial Communities

The least bacterial richness occurred in water runoff collected from CG, and the greatest from H (when control samples were excluded from the analysis). This result indicated that continuous grazing decreased bacterial richness in runoff, with no grazing increasing the bacterial richness in runoff samples (without control samples). This was not expected, as consistent grazing leads to less vegetative cover than other pasture management systems, thus causing greater bacterial movement and subsequent increased bacterial richness (Yang et al., 2020). Compared with non-agricultural samples, the bacterial richness was greater in pasture systems. This suggests bacterial richness in the oligotrophic river is lower than agricultural runoff samples. One explanation is that runoff samples from agricultural pastures included greater soil, soil bound nutrients, and manure sources, which are sources of greater microbial diversity (Ashworth et al., 2017; Yang et al., 2019).

Greater bacterial richness occurred in the runoff samples following poultry litter application to watersheds. Therefore, it is likely that poultry litter included sources of bacteria and introduced its own suite of bacteria to the watersheds, which had attenuating effects over time (Ashworth et al., 2017). Previous studies found similar results in that poultry litter applications increased bacterial richness in soil (Yang et al., 2019). Year effects also caused differences in runoff bacterial diversity, with greater diversity in 2019 than in 2018. However, no differences were found for bacterial richness between 2018 and 2019. Overall, the best management practices of grassed buffer strips and riparian buffers increased bacterial richness relative to overgrazed or continuously grazed systems, thus richness is improved by edge of field filter strips. In a previous study at this site, Pilon et al. (2018) found that these best management strategies increased carbon and nitrogen in long-term runoff, therefore, increased richness could be owing to greater microbial substrate (C and N) following these conservation practices. Such increases in runoff C, N, and richness could be due to greater plant mass in runoff from vegetative filter strips.

### Bacterial Community Composition Based on Pasture Management, Poultry Litter Application, and Sampling Year (With Control Samples)

Results illustrated the importance of evaluating water bacterial community across pasture management and sampling year, as well as the impact from poultry litter application timing. Beta diversity analysis indicated that bacterial community composition was different between runoff samples collected from our experimental watersheds and the control samples (Mulberry River, the National Wild and Scenic River). Water runoff samples included the dominant phylum Proteobacteria, however, the most abundance bacteria in control samples was Acidobacteria. Proteobacteria is a major phylum of Gram-negative bacteria including a variety of pathogenic genera, such as *Escherichia*, *Salmonella*, *Helicobacteria*, and many others (Madigan and Martinko, 2005). Proteobacteria has also been estimated as the most abundant phylum in most soils, where their functional roles are connected to such processes as nitrogen fixation and oxidation of iron, sulfur, and methane (Spain et al., 2009; Itavaara et al., 2016). Many studies also identified Proteobacteria as the dominant phylum in drinking water and sediment (Spring et al., 2000; Zhang et al., 2017). Considering the phylogenetic breadth of the Acidobacteria, it is similar to the metabolically diverse Proteobacteria, albeit they both fill different niches. Acidobacteria plays an important role in using nitrite as a N source, respond to soil macro-, micro-nutrients, and soil acidity, as well as expressing multiple active transporters, degrading gellan gum, and producing exopolysaccharide (Kielak et al., 2016).

The bacterial community composition was different pre- and post-poultry litter application, with diversity increasing directly following poultry litter land applications, which indicated that poultry litter was a possible source of bacteria and altered runoff community structure. The bacterial community structure also varied between 2018 and 2019, which indicated that bacterial communities in these watersheds may vary annually and interannually.

### Distribution of Four AMR Associated Genes in Runoff Samples Based on Pasture Management and Sampling Year

Overall, the main effect of pasture management (H, CG, R, RB, and RBR) had no effect on the four AMR-associated genes (*ermB*, *sulI*, *intlI*, and *bla_ctx-m-32_*) in water runoff samples (*p* > 0.05). Although there was a greater detection of *sulI* genes from runoff samples in the CG system in 2019, with H being the lowest in 2019. The *bla_ctx-m-32_* gene codes for beta-lactamase resistance and is one of the top AMR global health priorities. The *bla_ctx-m-32_* variant has been previously associated with cattle, cattle feces, and poultry (Cottell et al., 2013; Bevan et al., 2017; Palmeira and Ferreira, 2020), but was not detected in any of our post-poultry litter application runoff samples. A related study evaluating AMR associated genes in soils, indicated *ermB*, *sulI*, and *intlI* genes in soil were the highest under continuous grazing (relative to the conservation best management practices), suggesting overgrazing and continuous cattle manure deposition may increase AMR gene presence (Yang et al., 2020). In this same study, metagenomic shotgun sequencing revealed a greater total number of AMR genes in soils under long-term CG, while fewer AMR genes were found in H (no cattle manure; Yang et al., 2020). In the current study, we did not observe an increase in the macrolide resistance gene *ermB*, sulfonamide resistance gene *sul1*, or the integrase genes *intI1* in runoff based on pasture management. Of note, there was no amplification of these four genes from control water samples. Therefore, although bacterial community composition has been shown to impact bacterial resistomes in many habitats (Fosberg et al., 2014), the runoff bacterial community changes observed with different pasture management strategies was not linked with significant changes in the four AMR-associated genes quantified in this study. A study by Hall et al. (2020) found that a filter strip to surface water setback distance between 34 and 67 m is required to allow manure-borne antibiotics and ARGs in runoff to reach background levels; therefore, given that this experimental set up was less (<10 m) filter strip benefits on AMR gene reduction was not fully realized.

## Conclusion

Pasture management and animal manure additions had an influence on bacterial richness in water runoff, with the lowest bacterial richness occurring in CG and the greatest in H (when non-agricultural samples were excluded). These results could be attributed to a greater contribution of the poultry litter bacterial community in runoff in the absence of grazing, as more poultry litter remains on the soil surface and less incorporation occurs without cattle hooves acting as tillage in these systems. Further, bacterial richness was lower in control samples (Mulberry River, a National Wild and Scenic River) than experimental pasture watersheds. This suggests bacterial richness is greater in agricultural runoff samples. One possible explanation is that runoff samples from pastures included greater soil, soil bound nutrients, and manure sources, which are sources of microbial diversity. Finally, beta diversity was influenced by poultry litter application timing, with greater diversity occurring directly after poultry litter land applications, therefore it is likely that poultry litter introduced its own suite of bacteria to watersheds. Overall, conservation pasture management including grassed buffer strips and riparian buffers increased bacterial richness (Chao1) relative to “business as usual” or continuously grazed systems.

Long-term (14 years) pasture management resulted in detection of three AMR-associated genes (*ermB*, *sulI*, and *intlI*), but not in the non-agricultural (control) water body, which received runoff from soil, but not animal manure inputs (poultry litter). This suggests that runoff from recently applied poultry litter and cattle manure may act as sources of AMR-associated genes in runoff. Overall, these results highlight the importance of monitoring pasture management and poultry litter application timing on bacterial community analysis and AMR-associated genes in water runoff for the development of the best management or conservation agricultural strategies.

## Data Availability Statement

The datasets presented in this study can be found in online repositories. The names of the repository/repositories and accession number(s) can be found at: https://www.ncbi.nlm.nih.gov/, PRJNA717042.

## Author Contributions

AA received the funding, organized the experiment, and drafted the manuscript. YY conducted the laboratory and data analyses and developed the figures. LD, JD, and KC provided the guidance on analyses and data presentation. LD drafted the manuscript and assisted with data analysis. PM and PO contributed to the overall experimental management of long-term watersheds. MS provided the manuscript editing. All authors contributed to the article and approved the submitted version.

### Conflict of Interest

The authors declare that the research was conducted in the absence of any commercial or financial relationships that could be construed as a potential conflict of interest.

## Abbreviations

ARG: Antibiotic resistant genes
AMR: Antimicrobial resistance
PERMANOVA: Permutational analysis of variance
ANOVA: Analysis of variance
CG: Continuously grazed
H: Hayed system
RBR: Rotational grazing with a fenced riparian buffer
OTU: Operational taxonomic unit
PCoA: Principle coordinate analysis
qPCR: Quantitative-polymerase chain reaction
*ermB*: Erythromycin resistance gene
*sulI*: Sulfonamide resistance gene integrase gene
*intlI*: Integrase gene
*bla_ctx-m-32_*: *β*-Lactams resistance gene
